# Degradation kinetics and pathway of phenol by *Pseudomonas* and *Bacillus* species

**DOI:** 10.1080/13102818.2014.991638

**Published:** 2015-01-07

**Authors:** Syed Adnan Hasan, Suraiya Jabeen

**Affiliations:** ^a^ Department of Environmental Sciences, Sindh Madressatul Islam University Aiwan-e-Tijarat Road, Karachi-74000, Pakistan; ^b^ Institute of Environmental Studies, University of Karachi, Karachi-75270, Pakistan

**Keywords:** biodegradation, phenol, *Pseudomonas*, *Bacillus*, kinetics

## Abstract

This article elucidates that strain *Pseudomonas aeruginosa* (IES-*Ps*-1) is a versatile toxic organic compound degrader. With the degradation of malathion and cypermethrin (studied by other researchers previously), this strain was able to degrade phenol. Two other indigenous soil flora (i.e., *Pseudomonas* sp. (IES-S) and *Bacillus subtilis* (IES-B)) were also found to be potential phenol degraders.

Phenol was degraded with Monod kinetics during growth in nutrient broth and mineral salts medium. Before entering into the growth inhibition phase, strains IES-*Ps*-1, IES-S and IES-B could tolerate up to 400, 700 and 500 mg/L phenol, respectively, when contained in nutrient broth. However, according to the Luong–Levenspiel model, the growth of strains IES-*Ps*-1, IES-S and IES-B would cease at 2000, 2174 and 2190 mg/L phenol, respectively. Strain IES-*Ps*-1 degraded 700, 900 and 1050 mg/L phenol contained in mineral salts medium with the specific rates of 0.034, 0.075 and 0.021 h^−1^, respectively. All these strains grew by making clusters when exposed to phenol in order to prevent damages due to high substrate concentration. These strains transformed phenol into catechol, which was then degraded via *ortho*-cleavage pathway.

## Introduction

Being the basic structural units for a variety of organic compounds, phenol and its derivatives are used in oil refineries, manufacturing phenol and its derivatives, paints, pharmaceuticals, industries of resins, textile dyes, disinfectants, petrochemicals and paper mills, hence found in the effluents of these industries.[1–4]

Phenol is highly toxic to all life forms in all concentrations (5–2000 mg/L) and considered as a priority pollutant.[5,6] The daily average amount of phenol to human exposure should not exceed 20 mg.[1] Also, phenol can pose lethal effects in marine species at concentrations of 5–25 ppm.[4] Therefore, it is extremely important to develop efficient techniques to remove these pollutants from the environment to save natural ecosystems for sustainable development.[7]

A variety of methods are available for the removal of phenols from industrial effluents. The efficiency of these methods is mainly dependent on the time taken for the complete removal and the initial concentrations of phenols to be degraded. Some processes can be very costly and this is a large determinant when choosing a system. Adsorption of phenol on activated carbon,[8] solvent extraction [9] and chemical processes (i.e., chlorination, ozonation, benzol extraction and oxidation) can give up to 90% removal efficiency.[10,11] However, these processes usually produce a variety of intermediates and by-products, which are usually more toxic than the original substrate and lead to secondary effluent problems.[5,6,12] Toxic emissions from such processes can also be produced and these can be more damaging than the reactants.

Biological degradation is the most efficient and low-cost method for the removal of phenols from wastewaters [12–14] as it leads to complete mineralization of phenols and environmentally acceptable and less expensive.

Studies have shown that phenol can be aerobically degraded by a wide variety of microorganisms of genera *Pseudomonas*,[15–22] *Acinetobacter*,[23] *Alcaligenes*,[24,25] *Bacillus*,[26] *Burkholderia*,[27,28] *Nocardia*,[29] *Nocardioides*,[30] *Ralstonia* [31] and *Rhodococcus*.[32] In this study, we have presented the pathway and degradation kinetics of phenol by *Pseudomonas* and *Bacillus* species.

## Materials and methods

### Culture medium and growth conditions

Bacterial strains were grown aerobically at 35 °C under shake flask conditions. Growth medium (mineral salts medium, MM) contained per litre: 5.4 g of Na_2_HPO_4_⋅12H_2_O, 1.4 g of KH_2_PO_4_, 0.5 g of (NH_4_)_2_SO_4_, 0.2 g of MgSO_4_⋅7H_2_O, supplemented with trace elements solution (5 mL L^−1^) containing per litre 780 mg of Ca(NO_3_)_2_⋅4H_2_O, 200 mg of FeSO_4_⋅7H_2_O, 20 mg of Na_2_SeO_4_⋅10H_2_O, 10 mg of ZnSO_4_⋅7H_2_O, 10 mg of H_3_BO_3,_ 10 mg of CoCl_2_⋅6H_2_O, 10 mg of CuSO_4_⋅5H_2_O, 4 mg of MnSO_4_⋅H_2_O, 3 mg of Na_2_MoO_4_⋅2H_2_O, 2 mg of NiCl_2_⋅6H_2_O.

### Culture used

Three bacterial strains were used in this study. One of them was *Pseudomonas* sp*.* IES-*Ps*-1, which was initially isolated and identified in 1995 from soil using enrichment technique with malathion (an organophosphate pesticide) as the sole source of carbon and energy.[33] Later in 2004, strain IES-*Ps*-1 was used to study the degradation of cypermethrin (a common synthetic pyrethroid insecticide).[34] Since IES-*Ps*-1 showed high tolerance against hazardous chemicals, it was then used to degrade phenol in this study. The culture is characterized as motile, gram-negative and thin short rod shaped.

### Isolation of new bacterial strains able to degrade phenol

Two different species of phenol degraders were isolated and characterized by enrichment technique. For this, samples were collected from activated sludge of the treatment plant of a steel mill, which is situated at Karachi, Pakistan. Three test tubes containing 5 mL sterile nutrient broth (NB) were incubated with 1 g (wet weight) activated sludge and incubated under non-shaking conditions at 35 °C. Growth of microbial strains was observed after 24 h and samples of the enrichment culture were spread on nutrient agar (NA) plates. Morphologically different colonies from NA plates were separately incubated into 120 mL flasks containing 50 mL MM supplemented with 100 mg/L phenol. After every 7–8 days, cultures were inoculated into series of flasks containing 50 mL MM with increasing concentration of phenol (200–2000 mg/L). Phenol tolerant bacterial strains were isolated by streaking the loopful of MM on NA plates. Morphologically different strains were separately streaked on NA plates and preserved on NA slants, temporarily. The cultures were sub-cultured after every two–three months. Purity of the cultures was regularly checked during preservation and study period.

### Identification and characterization of isolated strains

Strain IES-*Ps*-1 was previously identified up to the genus level.[33] In this study, the specie type of this strain was identified. The genera and/or species of other isolates were identified by performing gram staining, motility, morphological characteristics and biochemical tests with some modifications described by Bergey's manual and Sivaraj et al.[35,36] Gram reactions and morphological characteristics of the isolated strains were observed under the oil immersion objectives of the microscope. True motility of the bacterial isolates was observed under high microscope magnification (1000X) using hanging drop method.

### Growth and degradation kinetics under batch conditions

For studying growth and degradation kinetics of isolated strains, pure cultures of strain IES-*Ps*-1, *IES-S* and *IES-B* were separately inoculated into a series of 500 mL Erlenmeyer flasks containing 250 mL NB and MM supplemented with 100–1200 mg/L of phenol. Flasks were incubated at 35 °C in a shaking water bath (Grant SS30, UK) at 125 rpm. Samples were drawn every hour from NB and after every day from the MM flasks. Growth kinetics of the strains was calculated by monitoring the optical densities at 600 nm and degradation kinetics were studied by measuring the residual concentration of phenol in the medium.

### Growth kinetics

For studying growth kinetics of *Pseudomonas* and *Bacillus* strains with phenol as the sole source of carbon and energy, mathematical modelling was performed. For this, we used the Monod equation:[37](1) 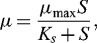
where µ is the specific growth rate (h^−1^), µ_max_ the maximum specific growth rate (h^−1^), *S* the substrate concentration (mmol/L) at time *t*, and *K_s_* is the half saturation coefficient (mmol/L).

For studying the growth inhibition of bacterial strains at high phenol concentrations, the Haldane–Andrew model [38,39] was used, which is given by the following equation:(2) 
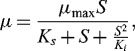
where *K_i_* is the substrate inhibition constant (mmol/L).

Luong [40] and Levenspiel [41] extended the Monod type model to describe the growth inhibition at high substrate concentrations:(3) 
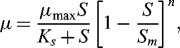
where *S_m_* is the critical inhibitor concentration above which growth stops and *n* is an empirical constant.

Growth and degradation rates of *Pseudomonas* and *Bacillus* strains on phenol were calculated by measuring the slopes of decreasing optical densities at 600 nm and phenol concentrations at different time points.

### Removal of phenol from the bioreactor

Degradation of phenol was studied by inoculating the respective bacterial strains in a bioreactor containing 4 litres of domestic wastewater collected from University of Karachi, Pakistan campus under non-sterile conditions. Physical conditions of the reactor were maintained (i.e., 35 °C, 350 rpm and normal pH) with constant supply of air in order to maintain 5 mg/L dissolved oxygen.

### Determination of cell concentration and electron microscopy of microbial strains

Cell concentrations of bacterial strains were determined from optical density measurements at 600 nm using a UV/visible spectrophotometer (Shimadzu, Japan) with the NB or MM as blank.

For visualization of bacterial strains, samples were prepared by taking 5 mL tryptic soy broth in corning tube. The desired culture was inoculated in the sterilized medium and incubated at 37 °C. After 24 h, 2 mL of 20% glucose solution was added in the culture medium and further incubated. All materials were discarded from corning tube after 24 h. Five millilitres of 99% methanol were added in the tube and left for 15 minutes, then discarded. Finally, 0.1% crystal violet was added and left for 20 minutes, then washed with sterilized distilled water. Air dried samples were cut into miniature pieces and placed under analytical scanning electron microscope (SEM).

The samples were plated with gold in JEOL quick auto coater JFC-1500 Ion Sputtering Device and observed with SEM (JEOL JSM – 6380, MP- 41080, Japan).

### Analytical methods

Residual concentrations of phenol were estimated with spectrophotometer (Shimadzu UV-Vis 1201, Japan) by 4-aminoantipyrene method.[22,42] and by reverse phase high pressure liquid chromatography (HPLC) using a Novapak C18 column (250 × 4.6 mm, 5 µm particle size). The mobile phase used was 100% methanol. An injection volume of 20 µL was used. The flow rate was 0.8 mL/min and detection at 254 nm was with a UV-Vis absorbance detector.

## Results and discussion

### Isolation of phenol degraders

Bacterial strains of different genera were found as potential degraders of phenol. These strains were separately streaked on agar plates containing varying concentrations of phenol. The concentration of phenol was gradually increased to check their tolerance. The highest concentration of phenol was 2000 mg/L. Finally, one gram positive Bacillus and two gram negative short rods were selected for studying the degradation of phenol.

### Identification and characterization of phenol degraders

The identification and characterization of bacterial isolates were performed using morphological, cultural and biochemical tests (Table 1) as described in Bergey's manual.[36] The identification of strain IES-*Ps*-1 was confirmed to be *Pseudomonas aeruginosa*. The other two new isolates were identified as *Pseudomonas* sp. and *Bacillus subtilis* and designated as IES-S and IES-B, respectively.

**Table 1.** Morphological, cultural and biochemical characteristics of isolated strains able to degrade phenol.

Test	IES-*Ps*-1	IES-S	IES-B
Description of colonies	Large, flat, greenish pigment	Pale coloured mucoid colonies on MacConkey	Large dry cream coloured irregular colonies on agar
Shape	Irregular	Regular	Irregular
Elevation	Umbonate	Convex	Flat
Gram reaction	Gram-negative short rods	Gram-negative short rods	Gram-positive short rods
Motility	Motile	Motile	Motile
O_2_ need	Strict aerobe	Strict aerobe	Strict aerobe
Optimal growth temperature (C)	37	37	37
Acid from glucose	+	−	+
Oxidase	+	+	−
Catalase	+	+	+
Indole	−	−	−
Citrate	+	+	−
H_2_S production	−	−	−
Nitrate reduction	+	−	+
Urea hydrolysis	+	+	−
Gelatin hydrolysis	+	+	+
Lactose fermentation	−	−	−
Sucrose	−	−	+
Fructose	+	−	−
Mannose	+	−	−
Ribose	+	−	−
Xylose	+	−	−
Cetramide agar base (CAB)	Fluorescent green colonies	−	−

### Kinetics of phenol degradation in NB

**Figure 1.**
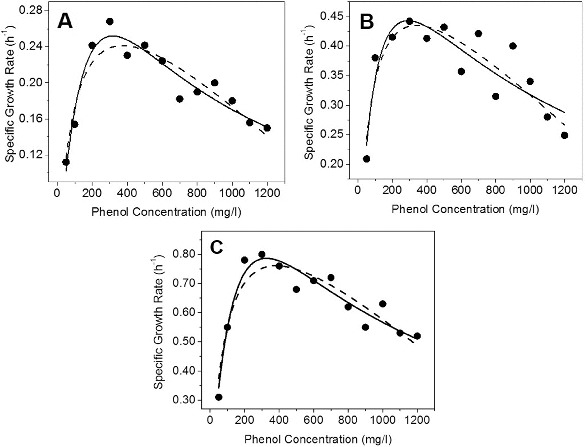
Growth kinetics of (A) IES-*Ps*-1, (B) IES-S and (C) IES-B in nutrient broth under shake flask conditions supplemented with phenol. Experimental data points are shown with dots. Haldane–Andrew regression curves are mentioned with regular lines and Luong–Levenspiel regression curves are indicated with dashed line.

In order to study the kinetic properties of *Pseudomonas* sp. strain IES-*Ps*-1, *B. subtilis* sp. strain IES-B and *Pseudomonas* sp. strain IES-S, we performed a number of growth experiments in batch cultures. Cells of these strains were separately incubated in NB containing increasing concentrations (50–1200 mg/L) of phenol (data not shown). A comparison in growth kinetics of strain IES-*Ps*-1 in two different media (enriched and artificial) was studied by incubating cells in separate flasks containing NB and MM supplemented with 100–1050 mg/L phenol. Cell growth with respect to time was measured in each batch culture with a certain phenol concentration. The specific growth rates at different phenol concentrations were calculated by measuring the slopes of increasing optical densities at 600 nm. From the results, it is obvious that the specific growth rate increases with an increase in substrate concentration until a maximum value is reached. With strain IES-*Ps*-1, IES-S and IES-B, the maximum specific growth rates were 0.26, 0.43 and 0.78 h^−1^, respectively (Figure 1). However, specific growth rates started to decrease when phenol concentrations were above 220, 310 and 320 mg/L in cultures of IES-*Ps*-1, IES-S and IES-B, respectively. This decline indicates the inhibition in growth, which can be caused due to cell damage or disruption of membrane integrity at higher phenol concentrations.[43,44]

In order to determine the growth parameters, the Monod model was used. The Haldane–Andrew and Luong–Levenspiel models were used to determine the effects of substrate inhibition on growth rates. Growth kinetic data were fitted by non-linear regression and the parameters of the kinetic constants were calculated with Microcal Origin 8.0. The parameters estimated by using these models are summarized in Table 2.

**Table 2.** Kinetic parameters obtained using Monod, Luong–Levenspiel and Haldane-Andrew models in batch mode with pure culture of *Pseudomonas* (IES-*Ps*-1), *Pseudomonas* (IES-S) and *Basillus* (IES-B) growing in NB supplemented with phenol.

	Strain IES-*Ps*-1			Strain IES-S			Strain IES-B		
Kinetic parameter	Monod model	Luong–Levenspiel model	Haldane–Andrew model	Monod model	Luong–Levenspiel model	Haldane–Andrew model	Monod model	Luong–Levenspiel model	Haldane–Andrew model
m_max_ (h^−1^)	0.30	0.38	0.60	0.51	0.63	0.8	0.9	1.2	1.8
*K_s_* (mg/l)	98	111	234	71	77	121	92	102	206
*K_i_* (mg/l)		–	394	–	–	696	–	–	520
*S_m_* (mg/l)		2000	–	–	2174	–	–	2190	–
*n*		1	–	–	1	–	–	1	–

The data of specific growth rate was fitted to Monod's model when the concentrations of phenol were lower than 400, 700 and 500 mg/L in strains IES-*Ps*-1, IES-S and IES-B, respectively. Haldane-Andrew and Luong–Levenspiel models were used when inhibition in growth started due to increase in phenol concentrations. The *K_s_* values in Monod's model indicate the capability of the isolates to grow at lower substrate concentrations. The values of *K_i_* in Haldane–Andrew model indicate the inhibition in growth of microbial strains that starts at a particular substrate concentration over which the specific growth and degradation rate decline. The higher *K_i_* (i.e., 696 mg/L) of strain IES-S and 520 mg/L of strain IES-B compared to lower value (i.e., 394 mg/L) of strain IES-*Ps*-1 indicated a higher tolerance level in growth.

In Luong–Levenspiel model, the value of maximum substrate concentration (*S_m_*) is used, at which the microbes cease to grow. The highest *S_m_* value was obtained in strain IES-B, indicating that this strain can grow with up to 2190 mg/L of phenol. Similarly, strain IES-*Ps*-1 and strain IES-S can survive and grow with up to 2000 and 2174 mg/L of phenol, respectively (Table 2).

### Kinetics of phenol degradation in mineral salts medium

**Figure 2.**
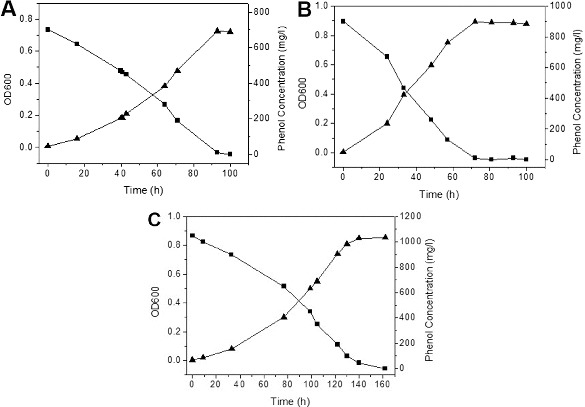
Growth kinetics of phenol degradation by *Pseudomonas* IES-*Ps*-1 in MM medium supplemented with phenol. (A) 700 mg/L, (B) 900 mg/L and (C) 1050 mg/L.

In order to study the growth kinetics of IES-*Ps*-1 and degradation kinetics of phenol in MM, we performed a number of batch experiments in shake flask conditions. Strain IES-*Ps*-1 grew exponentially between 16 and 93, 0 and 72 and 33 and 140 h with specific growth rates of 0.034, 0.075 and 0.021 h^−1^ when 700 (Figure 2A), 900 (Figure 2B) and 1050 mg/L (Figure 2C) of phenol was incubated in MM, respectively.

### Electron microscopy of phenol degraders

**Figure 3.**
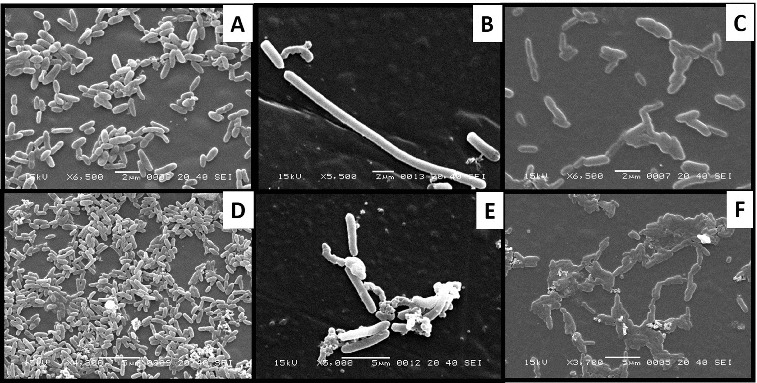
Cellular morphology of (A) *Pseudomonas* sp. IES-*Ps*-1 at 6500X magnification, (B) *Bacillus* sp. IES-B at 5500X magnification and (C) *Pseudomonas* sp. IES-S at 6500X magnification when grown in nutrient broth and the respective cells of IES-*Ps*-1, (D) IES-B (E) and IES-S (F) grown in mineral medium supplemented with phenol.

The cultural morphology of bacterial isolates was studied by electron microscopy. The SEM images reveal that the isolated strains are rod-shaped cells with approximate 1 1 μm mu;m in width and 2–4 4 μm mu;m in length (Figure 3). It was also observed that the cells growing in plain NB were scattered. However, cells growing in NB containing phenol formed clusters in order to prevent damages due to high phenol concentration and the metabolites, which are formed intermediately. This behaviour of isolated strains can be explained with the fact that cluster formation is a type of defence mechanism in several microbial strains. Proteins, which are required to mineralize a substrate are mainly expressed under normal growth conditions. However, several other proteins (i.e., chaperonin, succinyl-CoA synthetase-*β*-subunit, thioredoxin reductase and superoxide dismutase) are also expressed that help cells growing under stress (i.e., high substrate concentration in this study).[45–47]

### Degradation pathway of phenol

**Figure 4.**
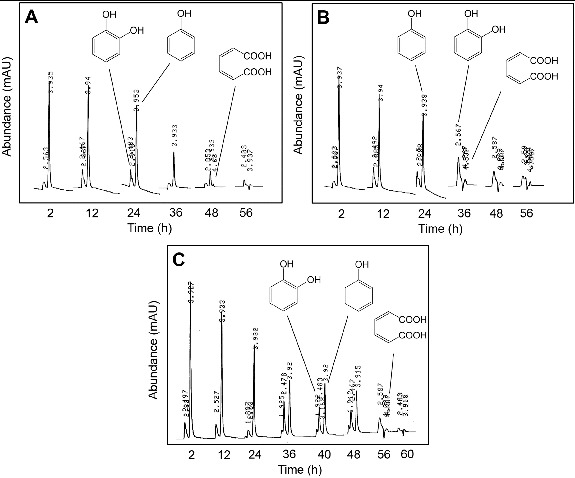
HPLC chromatograms showing the degradation of phenol in a bioreactor under non-sterile conditions supplemented with 700 mg/L of phenol when the bioreactor was inoculated with the cells of (A) *Pseudomonas* sp. strain IES-*Ps*-1, (B) *Bacillus* sp. strain IES-B and (C) *Pseudomonas* sp. strain IES-S.

Degradation of phenol in MM by *Pseudomonas* sp. strain IES-*Ps*-1 in shake flask conditions was very slow during the first 24 h (Figure 4(A)). During this period, a peak of catechol with increasing concentration was observed through HPLC. After 24 h, quick degradation of phenol occurred and catechol disappeared. This indicates that the enzyme required for the degradation of phenol (i.e., phenol hydroxylase) was not as active as the enzyme that transforms catechol (i.e., catechol 1, 2-dioxygenase). Later, trace of *cis*, *cis*-muconate was observed when over 95% phenol and 99% catechol were degraded.

Similar behaviour in *Bacillus* sp. strain IES-B was observed during degradation of phenol. A very slow degradation of phenol in the first 24 h was observed with the accumulation of trace amount of catechol (Figure 4(B)). A rapid degradation of phenol and catechol was observed in subsequent hours. Complete degradation of phenol occurred after 36 h and trace of *cis,cis*-muconate was monitored.

*Pseudomonas* sp. strain IES-S degraded phenol very slowly in the first 24 h (Figure 4C). Later, the concentration of phenol decreased quickly and became constant for 12 h with the accumulation of catechol. Phenol was completely degraded after 56 h with the accumulation of traces of catechol and muconic acid, which disappeared after 60 h.

**Figure 5.**
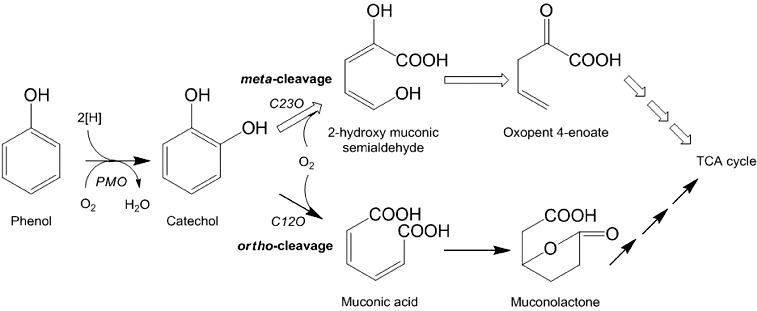
Phenol degradation pathway. The enzymes involved in first two steps of degradation are, PMO = phenol monooxygenase, C12O = catechol 1,2-dioxygenase and C23O = catechol 2,3-dioxygenase.

Degradation studies of phenol by various *Pseudomonas* species have been conducted by many researchers under aerobic and anaerobic conditions.[19,20,48–53] The findings of this study suggest that phenol is aerobically degraded in MM by the isolated strains through *ortho*-cleavage pathway. The degradation is initiated with the formation of catechol, in which phenol hydroxylase (a monooxygenase) attached a hydroxyl group at *ortho* position with the benzene ring. Catechol is the main intermediate formed during biodegradation of phenol by different microbial strains, [54] which is degraded either via *meta*-cleavage by catechol 2,3-dioxygenase [13,55] or *ortho*-cleavage by catechol 1,2-dioxygenase.[56] The transformation of catechol via any route occurs very quickly by dioxygenases that results in quick disappearance of the immediate metabolites (i.e., *cis*, *cis*-muconate through *ortho*-cleavage or 2-hydroxymuconic semialdehyde through *meta*-cleavage).[19,48,49] In this study, we observed the formation of muconic acid with all three isolates (Figure 4), which indicates that phenol degradation occurs through an *ortho*-cleavage pathway. Later, the metabolites enter into the tricarboxylic acid cycle for complete degradation of phenol (Figure 5).

## Conclusion

The results obtained through this study indicate that the isolated strains IES-*Ps*-1, IES-S and IES-B degraded phenol through an *ortho*-cleavage pathway. These strains possess high tolerance to phenol toxicity and are capable to degrade up to 400, 700 and 500 mg/L phenol, respectively, without any significant inhibition in batch conditions, hence could be utilized as capable candidates for bioremediation of phenolic wastewaters.

## Acknowledgements

The authors would like to thank Mr Aamir Alamgir for proof reading the manuscript.

## Disclosure statement

No potential conflict of interest was reported by the author(s).

## Funding

The authors acknowledge the financial support from office of the dean faculty of science, University of Karachi through project grant invoice [NO. DFS/2007-162].
